# Metabolic Reprogramming and the Recovery of Physiological Functionality in 3D Cultures in Micro-Bioreactors †

**DOI:** 10.3390/bioengineering5010022

**Published:** 2018-03-07

**Authors:** Krzysztof Wrzesinski, Stephen J. Fey

**Affiliations:** 1 Tissue Culture Engineering Laboratory, Department of Biochemistry and Molecular Biology, University of Southern Denmark, 5230 Odense, Denmark; kwr@celvivo.com; 2 CelVivo IVS, 5491 Blommenslyst, Denmark

**Keywords:** bioreactors, 3D cell culture, spheroids, organoids, hypoxia, aerobic glycolysis, glutaminolysis, metabolic reprogramming, physiological performance, Warburg

## Abstract

The recovery of physiological functionality, which is commonly seen in tissue mimetic three-dimensional (3D) cellular aggregates (organoids, spheroids, acini, etc.), has been observed in cells of many origins (primary tissues, embryonic stem cells (ESCs), induced pluripotent stem cells (iPSCs), and immortal cell lines). This plurality and plasticity suggest that probably several basic principles promote this recovery process. The aim of this study was to identify these basic principles and describe how they are regulated so that they can be taken in consideration when micro-bioreactors are designed. Here, we provide evidence that one of these basic principles is hypoxia, which is a natural consequence of multicellular structures grown in microgravity cultures. Hypoxia drives a partial metabolic reprogramming to aerobic glycolysis and an increased anabolic synthesis. A second principle is the activation of cytoplasmic glutaminolysis for lipogenesis. Glutaminolysis is activated in the presence of hypo- or normo-glycaemic conditions and in turn is geared to the hexosamine pathway. The reducing power needed is produced in the pentose phosphate pathway, a prime function of glucose metabolism. Cytoskeletal reconstruction, histone modification, and the recovery of the physiological phenotype can all be traced to adaptive changes in the underlying cellular metabolism. These changes are coordinated by mTOR/Akt, p53 and non-canonical Wnt signaling pathways, while myc and NF-kB appear to be relatively inactive. Partial metabolic reprogramming to aerobic glycolysis, originally described by Warburg, is independent of the cell’s rate of proliferation, but is interwoven with the cells abilities to execute advanced functionality needed for replicating the tissues physiological performance.

## 1. Introduction

Three-dimensional (3D) cell culture offers a glimpse into tissue function that is only recently being appreciated. This is true whether primary, immortal or stem cells are used and it appears to apply to all types of tissue.

What drives this process? Often the growth conditions used for these two-dimensional (2D) or 3D cell culture studies are identical: the same growth media, temperature, and atmosphere. Often the same cells are used and yet the performance of the cells is radically different. Here we examine the role of metabolic reprogramming and factors that induce the recovery or development of mimetic tissues.

Primary cells retain their physiological behaviour longer when grown in 3D culture conditions (e.g., astrocytes [1], prostate [2], and microvascular networks [3]). Numerous different colorectal cancer-derived tumour spheroids retain characteristics of original tumours [4,5] and lung cancer spheroids their chemosensitivity [6] but cardiac pluripotent cells do adapt to growth in 3D culture [7]. Immortal cell lines recover (‘re-differentiate’) structural and functional features of their parental tissue and regain in vivo drug sensitivity (e.g., breast, MCF-7 [8,9]; pancreatic β-cell β-TC6 [10] and RIN 5F [11]; glial-like GL15, neuronal-like SH-SY5Y [12]; ovarian, OV-MZ-6 and SKOV-3 [13]; trophoblast BeWo, Jeg-3 and JAr [14]; and liver, HepG2 [15], HuH-6 [16], HepG2/C3A [17,18,19]. Stem cells, whether embryonic or induced pluripotent, differentiate into 3D organoids when provided with appropriate molecular guidelines (e.g., ECM and growth factors) [20,21]. These recapitulate differentiation into a wide variety of mimetic tissues. (e.g., optic cup [22], pancreas [23] & gastric [24]). Growth in 3D microenvironments boosts the induction of pluripotency [25]. Transplantation of 3D structures into animals induces them to differentiate further, in some cases, to tissues that are almost indistinguishable from the native organ [26]. 

While the term ‘spheroid’ is usually used to indicate a mimetic tissue that is constructed from immortal cells and the term ‘organoid’ used to indicate a mimetic tissue derived from primary cells (including stem cells of any derivation) the basic principles driving recovery will be the same and so for the purposes of this article, the word ‘spheroid’ is used for both. 

**Figure 1:**
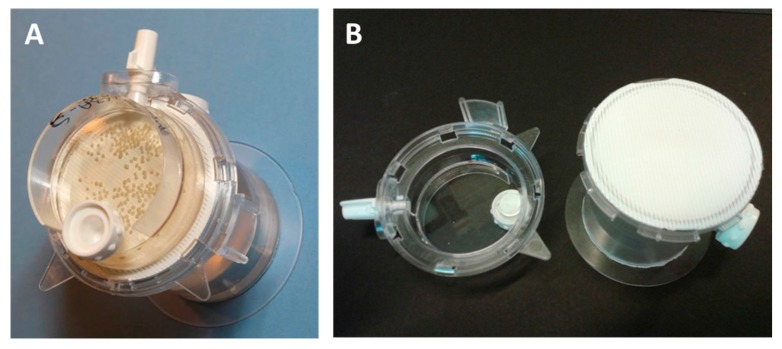
(**A**) an assembled bioreactor containing >300 21 day old spheroids. (**B**) The open bioreactor with (left) a 10 mL petri-dish like culture chamber and (right) the gas exchange membrane. Behind the membrane is a water reservoir and humidification labyrinth. White stoppers allow access for media change or filling the reservoir. This type of bioreactor has a gas membrane exchange area of 13.2 cm^2^ and a fixed volume (nominally 10 mL) and is available from CelVivo (Denmark).

Clinostat rotating vessels (Figure 1, also known as rotating wall vessels (RWV), rotating cell culture systems (RCCS) or high aspect rotating wall vessels (HARV)), are commonly used to generate a ‘microgravity’ environment that is conducive to the production of highly reproducible long lived 3D cultures which allow for the investigation and manipulation of mimetic tissues. Strictly speaking, they produce omnidirectional gravity—i.e., the tissue is influenced by gravity from all sides, effectively neutralizing directional gravity effects. In practice, the gravity is slightly larger than 1G: a clinostat running at 20 rpm will generate a G-force of 1.0089 at 2 cm from the axis of rotation.

The clinostat bioreactor has a number of advantages. Most cells in tissues, with the obvious exception of endothelial cells, experience little or no shear forces. Critical/lethal shear stresses for different mammalian cell types are in the range of 0.3–1.7 Pascal (1 Pa = 10 dyne/cm^2^) (Croughan and Wang, 1991) [27].

Mimetic tissue culture in a clinostat bioreactor provides very low shear forces (at 20 rpm, ca. 0.01 Pa [28] on the suspended spheroids, similar to rocking platform ‘wave’ bioreactors (set at an oscillation of 7° at 20 rpm) [29,30] and 0.02–0.064 Pa for micro-fluidic devices (flow rate 650 μL/min) [31,32]. Higher shear forces (and cellular effects) are seen for stirred suspension bioreactors (100–200 rpm, 0.3–0.66 Pa) [33,34] and for orbital shakers (20–60 rpm, 0.6–1.6 Pa) [35,36].

In rocking platform bioreactors and micro-fluidic devices, the mimetic tissue is usually in contact with plastic surfaces and the shear forces vary either with time, location, or both. This will induce differential growth rates, sizes and biochemical properties of the spheroids [37]. These differences will affect, for example, drug response [38]. The clinostat bioreactor exposes all spheroids to an equal and very low shear force and has been show to result in uniform spheroids that even after 21 days in culture have a standard deviation in their size of only ±21%. Clinostat spheroids are therefore the best suited for studies of metabolism or pharmacology, especially for kinetic measurements.

This recovery of the physiological phenotype in 3D culture suggests that either there is a common driving mechanism or that it occurs spontaneously. Strangely enough, clues as to why this happens can be found in ideas that have been around for almost 100 years.

### 1.1. The Relationship between Oxidative Phosphorylation and Aerobic Glycolysis

Rapid cancer cell proliferation favours reprogramming from oxidative phosphorylation to aerobic glycolysis. This concept was described initially by Warburg in the 1920’s [39,40]. He observed that proliferating ascites tumour cells convert most of their glucose to lactate and referred to this process as aerobic glycolysis because it occurred regardless of whether oxygen was present or absent [41].

In dynamic nuclear polarization (DNP) spectroscopic techniques, hyperpolarized [^13^C]-labelled pyruvate or other glycolytic intermediates have shown that tumours in situ produce lactate at levels which correlate with their degree of tumour progression or response to treatment [42,43]. Similar studies using hyperpolarized [^13^C]-labelled glucose revealed increased lactate production in mouse lymphoma and lung tumours, but not in healthy tissues [44]. Other whole-body approaches, such as PET and MRI brain-imaging techniques, have measured that 10–15% of the glucose that is used by the healthy brain is metabolised by aerobic glycolysis [45]. Similar results have been obtained in vitro using perfused heart and liver tissues [46] and in cell culture, comparing non-proliferating myocytes with proliferating Rh30 cell line. The proportion of aerobic glycolysis to oxidative phosphorylation varies between different tumour types: rapidly growing tumours tending to utilise a higher degree of aerobic glycolysis, in keeping with their reduced reliance on oxygen and their need to synthesise larger amounts of precursors faster [40,47].

Warburg hypothesized that metabolic reprogramming was specific to cancer cells, and that it arose from mitochondrial defects [39]. While his observations have been corroborated many times, the hypothesis that cancer growth was driven by these defects has been disproven [40,48,49]. Weinberg et al. demonstrated that mitochondrially generated reactive oxygen species are essential for Kras-induced proliferation and tumourigenesis of HCT116 colon cancer cells [50].

The current viewpoint is that, in aerobic glycolysis, glucose is metabolised via the glycolytic pathway to produce lactic acid, nucleotides, amino acids, and other metabolites. Simultaneously, glutamine is converted via glutaminolysis to citrate for cholesterol and lipid production. In contrast, non-proliferating, differentiated cells in healthy tissues efficiently produce ATP through oxidative phosphorylation. In oxidative phosphorylation, glucose is metabolised via the glycolytic pathway and then the pyruvate produced is oxidised to CO_2_ and water in the tricarboxylic (TCA) cycle. The electrochemical gradient generated is used to produce ATP [41].

### 1.2. Are Growth Rates Inversely Related to Functionality?

Although generally accepted, there is a flaw in the argument that rapidly growing cells preferentially utilise aerobic metabolism. Most tumours and healthy tissues proliferate relatively slowly, doubling their numbers every 20–60 days [51]. In contrast, cultured cells double every 1–8 days and therefore would be expected to exhibit strong aerobic metabolism. This is not the case. In a painstaking review, using oxygen consumption and lactate production to define oxidative and glycolytic ATP production, Zu and Guppy showed that there is no evidence indicating that cancer cells or cells in culture are inherently glycolytic [52]. Despite considerable variation, both normal and cancer cells produce about 19% of their ATP using aerobic glycolysis and the rest by oxidative phosphorylation. They concluded that cancers tend to be glycolytic because they are hypoxic [52]. Metabolic reprogramming to aerobic glycolysis has been called a hallmark of cancer [41,53,54], but in practice, it is a consequence of hypoxia.

There is another paradox. By definition, healthy tissues exhibit full physiological functionality. Tumours tend to lose these functionalities in a reciprocal proportion to their proliferation rate [51,55,56]. Immortal cell lines are often considered a ‘terminal condition’ for tumour cells where they proliferate rapidly but have lost much of their in vivo functionality. However, when immortal cells are given sufficient time to adapt to 3D conditions, their proliferation rate slows to that seen in tumours and healthy tissues in vivo and they regain physiological functionality [18,19].

So, the question becomes how do growth rate, metabolic reprogramming, and physiological functionality relate to each other. Is proliferation linked to metabolic reprogramming or is it independent? Is there a ‘spectrum’ between, on the one extreme, healthy tissue and at the other, immortal cell lines grown in 2D? Or are the ‘axes’ of normal to transformed independent of the axis of hyperoxic to hypoxic? Where should 3D cultures ‘be placed’ in this spectrum? Does metabolic reprogramming to aerobic glycolysis invariably lead to the loss of physiological function, as seen in the transformation of normal tissue to cancer, or are these phenomena independent? Can cells reprogram between oxidative phosphorylation and aerobic glycolysis based on their growth requirements or does it have consequences? We have addressed these questions by reviewing what is known about metabolic reprograming.

## 2. Materials and Methods

This manuscript is based on the deeper evaluation of raw data published previously as supplementary data [57]. For convenience, we describe here in brief the methods used. A full description can be found in our previous manuscript.

### 2.1. Cell Culture

HepG2/C3A cells were grown in DMEM containing 1 g glucose/L. In 2D conditions, they were left until they were nearly confluent (day 5) before they were collected for mass spectrometry. HepG2/C3A cell spheroids were prepared using AggreWell™ 400 plates (Stemcell Technologies, Vancouver, Canada) and left to mature in a rotating ‘microgravity’ micro-bioreactors on a BioArray Matrix drive (CelVivo IVS, Blommenslyst, Denmark) for at least 21 days to reach a dynamic equilibrium [57]. The clinostat 3D culture reduces shear forces on the cells to a minimum while increasing nutrient and gas exchange (see Supplementary video).

### 2.2. Determination of Protein Content of Spheroids

Spheroids were washed with PBS, collected, photographed to calculate their individual protein content using a look-up table derived, as described previously [17].

### 2.3. Determination of Glucose and Glycogen Content of Spheroids

Glucose in the media was measured using a Onetouch Vita glucose meter’ and test strips, (LifeScan, Inc., Cat Nos. 6407078 and 6407079 respectively, Milpitas, CA, USA). Total glycogen was measured in individual spheroids using a flurometric assay kit (Sigma cat. No. MAK016, Merck KGaA, Darmstadt, Germany). Glycogen is hydrolysed to glucose and measured in a fluorometer. Amounts of glucose in the spheroids prior to glycogen hydrolysis were negligible.

### 2.4. Mass Spectroscopy

Protein samples were collected from classical 2D cell culture five days after trypsinisation and from 3D spheroid culture 21 days after spheroid culture initiation. The proteins were quantitated, alkylated, digested with trypsin and washed, stable-isotope dimethyl labelled, and electrosprayed into the LTQ Orbitrap Velos (Thermo Scientific, Waltham, MA, USA). Data on 1346 proteins was deemed statistically reliable and was analysed with reference to multiple programs and information sources including MedLine, SwissProt, Kegg, Ingenuity™ and Go protein annotations [57].

## 3. Results and Discussion

We previously catalogued a plethora of differences between growing HepG2/C3A cells in 2D and 3D conditions. These include changes in the cell architecture (actin, microtubules, intermediate filaments) and metabolism (glycolysis, fatty acid metabolism, cholesterol and urea synthesis, DNA repair, RNA processing, protein folding and degradation, cell cycle arrest, transport around the cell) [57]. In this manuscript we take the analysis of the raw data a step further and use it to describe a coherent model of the driving force behind these differences between 2D and 3D culture. Where the cell type used to construct the spheroids is not defined in this article, the results refer to the raw data generated with HepG2/C3A.

### 3.1. Adaptation to Growth in 3D Culture

Cells need time to adapt to growing in 3D cultures and implement the changes catalogued above: to slow growth rates, reorganise their cytoskeleton, establish tight junctions, polarity, relocate membrane transporters and secrete tissue specific extracellular matrix components. Establishment coincides with the time needed for spheroids to reach a radius where their core is severely hypoxic. In a passive diffusion culture system (ultra-low attachment dishes or hanging drop) this requires about 8 days. Using NIH 3T3 fibroblasts or HepG2/C3A hepatocytes, the spheroid’s radius is about 160 µm [58,59]. In irrigated spheroids, (i.e., where media flows past them in microgravity cultures), this occurs after 18 days (radius is 450 µm) [18].

Evidence for hypoxia-induced metabolic reprogramming to aerobic glycosylation has been provided by reanalysing proteomic studies of cells grown in 2D and clinostat 3D conditions. Protein abundance has been measured in mature, 21 day old spheroids and compared to that seen in 80% confluent, five day old 2D cultures of the same cells (human hepatocellular carcinoma HepG2/C3A) using quantitative proteomics [57] (the raw data is presented in supplementary information) and [60]. For convenience, the reciprocal has been taken of all values below 1 (i.e., where the protein level is lower in 3D than in 2D), and this is indicated with a negative sign. In this way, equally significant changes, for example, a doubling or a halving of the amount of protein would be indicated by ‘2.00’ or ‘−2.00’ respectively.

**Figure 2:**
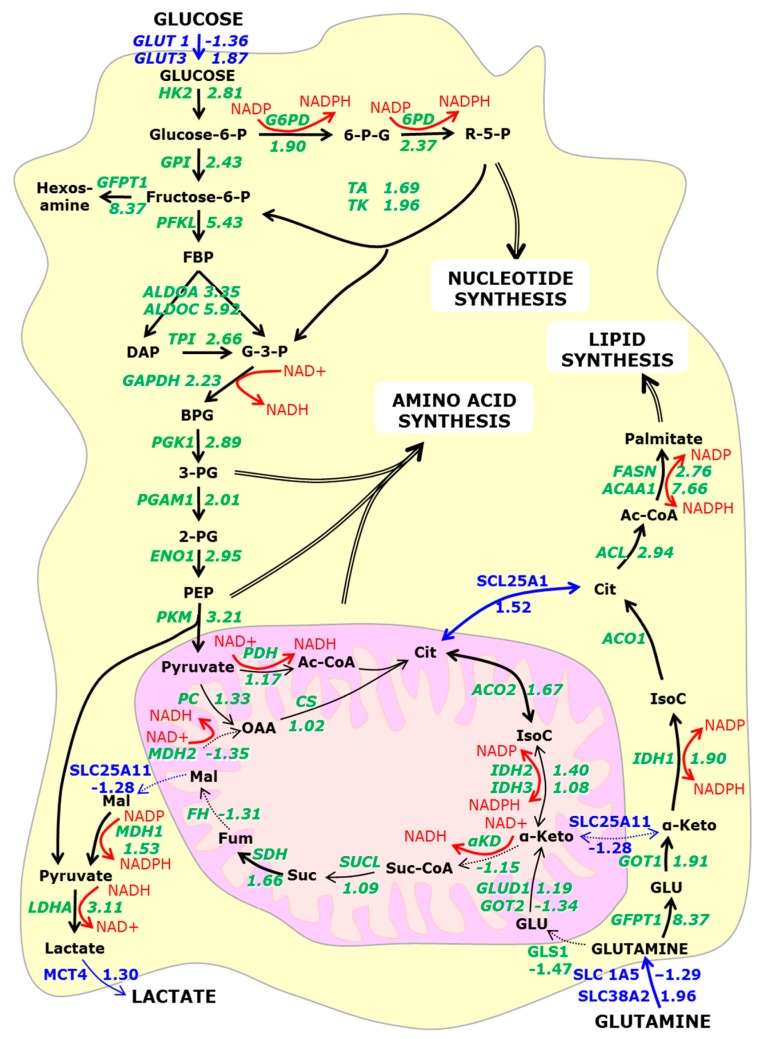
Ratios of the protein abundance of central metabolic enzymes and membrane transporter in 3D compared to two-dimensional (2D) cultures. Metabolites are marked in black, enzymes in green, transporters in blue and selected cofactors in red. Negative ratios indicate that the protein is present in higher amounts in 2D cultures. Arrows connecting metabolites are marked in bold if the enzyme expression is increased by a factor of 1.5 or greater. Arrows connecting metabolites are dotted if the enzyme expression is decreased. (Raw data taken from [57]).

The central metabolic pathways are illustrated in Figure 2. Enzyme expression levels illustrate a clear increase in glycolytic, glutaminolytic, hexosamine, and pentose phosphate pathways, as well as increased nucleotide, amino acid, and lipid synthesis. In contrast, enzymes of the TCA cycle are essentially unchanged. This is consistent with metabolic reprogramming [58,61]. If enzyme levels can be used to roughly indicate enzymatic activity, then one third of the glucose is metabolised by oxidative phosphorylation, corresponding to that seen in a rapidly growing tumour.

Metabolic reprogramming renders cancer cells susceptible to growth suppression because of their increased dependence on glucose for these anabolic pathways. Tumour cells predominantly express the embryonic M2 splicing isoform of pyruvate kinase (PKM2) [62]. Short hairpin RNA knockdown of PKM2 leads to its replacement by the adult PKM1 form, reverses metabolic reprogramming, increasing oxygen consumption and reducing lactate production and tumourigenicity in nude mouse xenografts [63]. PKM1 and PKM2 switching is regulated by three heterogeneous nuclear ribonucleoproteins hnRNPA1, hnRNPA2 and the polypyrimidine tract binding protein PTBP1 (or hnRNPI). These proteins bind flanking regions around exon 9, and in doing so, promote PKM2 expression [64]. In 3D spheroids, their expression is reduced (hnRNPA1 −2.20; hnRMPA2 −2.29; PTBP1 −1.23) favouring PKM1 and oxidative phosphorylation. However, no PKM1-specific peptides were detected in the original mass spectrometry data making it impossible to differentiate them [57].

Metabolic reprogramming results in the cell becoming increasingly dependent on glutaminolysis for fatty acid synthesis [41,58,61]. The key enzyme, ATP citrate lyase (ACL) shows increased expression in tumours (lung, prostate, bladder, breast, liver, stomach and colon) [65] and in spheroids (Figure 2). Cytoplasmic isocitrate dehydrogenase 1 produces isocitrate from α-ketoglutarate. IDH-1 mutations have been associated with gliomas via nuclear factor-κB activation in an hypoxia-inducible-factor (HIF1-α) dependent manner [66]. Inhibition of the IDH-1 or ACL inhibits A549 metabolism in vitro and, when injected into nude mice, reduces tumour growth [49,58].

Interestingly, activated effector T cells (TE) utilise anabolic aerobic glycolysis, while memory T cells (TM) use catabolic pathways. Microscopy shows that TE cells have punctate mitochondria, while TM cells maintain fused networks. The protein Opa1 is required for maintaining fused networks [67]. Opa1 is reduced in 3D spheroids (OPA1 −2.71), also indicating that they utilise aerobic glycolysis [18].

Evidence for metabolic reprogramming of cells in culture has also been found using hyperpolarized [^13^C] spectroscopy. Two cell lines (Huh-7 hepatocellular carcinoma cells and SF188-derived glioblastoma cells) have been cultivated in 2D and pulse-labelled with hyperpolarized [1-^13^C] pyruvate to determine the activities of pyruvate dehydrogenase (PDH, as a surrogate indicator of oxphos) and pyruvate carboxylase (PKM, for lactate). While both enzymes were active, supplementation with glucose favoured lactate production. Inhibition of glycolysis using an Akt inhibitor reversed this effect [68]. This illustrates that cells can adapt their metabolic activity to their environment. Rat hepatoma cells (JM1) have been probed using [^13^C]-labelled glucose while cultivated in either 2D or 3D conditions (encapsulated in alginate beads). These studies showed that in both conditions, 85% of [^13^C]-glucose was converted either to lactate or alanine by aerobic glycolysis [69].

Jiang et al., compared the metabolic activity of human H460, A549, MCF7, and HT-29 cells grown in 2D and 3D cultures [1-^13^C] glutamine or [5-^13^C] glutamine tracers clearly illustrated that citrate and lipids predominantly were synthesised via reductive glutaminolysis. In particular, while neither isocitrate dehydrogenase-1 nor -2 (cytosolic or mitochondrial, respectively) were necessary for monolayer growth, spheroids were dependent on the cytosolic IDH1 for glutaminolysis. Many cell lines (lung, mammary, colon, embryonic fibroblasts, squamous cell carcinoma, melanoma, glioblastoma, and leukaemia) use glutamine as their primary source of acetyl-CoA for lipogenesis [50,70]. Glycolytic ATP is only necessary in hypoxic conditions [37] and glutamine consumption is increased by reducing the available oxygen to 1% [61]. In these conditions, the major role of glucose metabolism is to drive the pentose phosphate pathway to generate NADPH. Glutaminolysis drives acyl-CoA production and lipidogenesis [50].

### 3.2. Is Metabolic Reprogramming Driven by Oxygen or Glucose Insufficiency?

Given that spheroids constructed from many types of cell exhibit metabolic reprogramming, the question arises as to what causes the switch?

#### 3.2.1. Diffusion Gradients and the Importance of Irrigation

Mammalian cells need oxygen and nutrients. In tissues, they are normally located within 100 to 200 µm of capillaries [71]. This corresponds to roughly 10–40 cell layers thick. An experimentally derived diffusion limit (i.e., where the PO_2_ level falls to 0) of 232 ± 22 µm agrees well with this [72]. Many cells in tissues experience low oxygen tension (e.g., 1% O_2_) [73] and it is alternative stressors, such as serum deprivation or acidosis, which induce cell death [74]. Only severe hypoxia (<0.01%) O_2_ is capable of inducing apoptosis [75].

**Figure 3:**
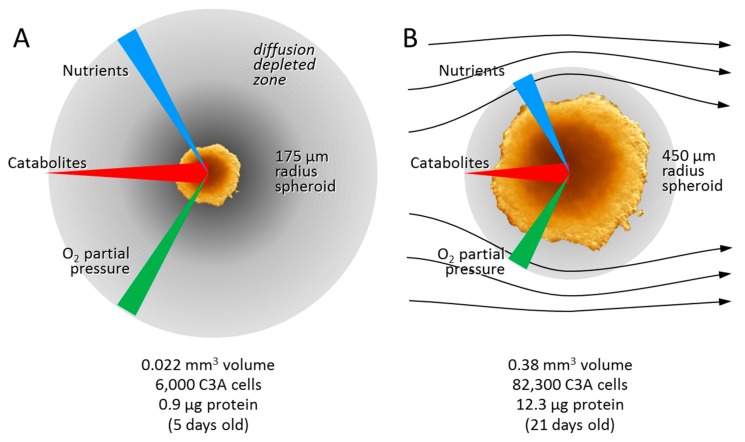
The diffusion depleted zone and its consequences for the oxygen, nutrient and catabolite gradients in (**A**) passive and (**B**) irrigated 3D culture. The size shown indicates the approximate maximum size above which anoxia develops in the spheroid core. Volumes, number of cells and amounts of protein are indicated for each. The apparent increase in cell volume is attributed in part to increased ECM and the development of sinusoidal and bile cannalicular spaces between the cells.

The most significant differences between 2D and 3D culture are diffusion gradients. Several types exist including gasses, nutrients, metabolites, signalling molecules, secondary messengers and growth factors. Here we will only consider oxygen, CO_2_ and glucose, since they have been suggested to drive metabolic reprogramming and need to be taken into consideration when designing micro-bioreactors (Figure 3).

The existence and depth of the hypoxic zone depends on several factors, including radius, cell type, and media flow rate. Measured diffusion gradients for oxygen follow smooth sigmoidal curves (with no difference in shape outside or inside EMT6 spheroids), suggesting that the presence of cells has little influence on its diffusability. Atmospheric oxygen (21%) provides a partial pressure (PO_2_) of about 145 mm Hg (ca. 190 µM) in media. In static cultures where there is no flow of plasma or media, the PO_2_ falls rapidly towards the spheroid’s centre (Figure 3A). Small radius spheroids (25–50 µm) have about 3.3% PO_2_ in their core, close to physiological levels in the brain. 100 µm radius spheroids have about 1.6% PO_2_ and larger spheroids less [76]. In irrigated spheroids, where media flows past the spheroid, the PO_2_ falls to about 13% measured in the media at the surface of the spheroid [77]. Thus, even when irrigated, there is a ‘diffusion-depleted zone’ in the media surrounding each spheroid (grey zones in Figure 3). The PO_2_ reaches a minimal plateau of 3.3% at about 150 µm into a 352 µm radius irrigated EMT6 spheroid, and 1.6% at about 225 µm into a 480 µm radius spheroid. In the latter case, stopping the media flow causes the core PO_2_ to quickly fall to 0. Doubling the flow rate had a marginal effect and was confined to the spheroid surface [78]. Core PO_2_ reached 0% in irrigated spheroids with radii greater than 600 µm.

Haemoglobin is normally found in hepatocytes and several cell lines. Hepatocarcinoma spheroids increase their haemoglobin content by a factor of thirty to actively alleviate low PO_2_ oxygen concentrations [57].

Experiments using pH as a spatial readout have demonstrated that the diffusivity of CO_2_ through spheroids (colorectal HCT116 and HT29, breast MDA-MB-468, pancreatic MiaPaca2, cervical squamous cell carcinomas HeLa and SiHa and ovarian clear cell adenocarcinoma OVTOKO) is exactly the same as its diffusivity through water (2.5 × 103 µm^2^/s) [79]. Usually, the spheroids’ cores are slightly more acidic than the surrounding media, possibly due to increased CO_2_ or lactate amounts [80,81].

No data is available describing a glucose gradient in or around spheroids. When considering that the glucose molecule is larger than oxygen or CO_2_, the gradient would intrinsically be expected to be steeper, but glucose transporters may alleviate this.

#### 3.2.2. Hypoxia Affects Glycolysis and Oxidative Phosphorylation

**Figure 4:**
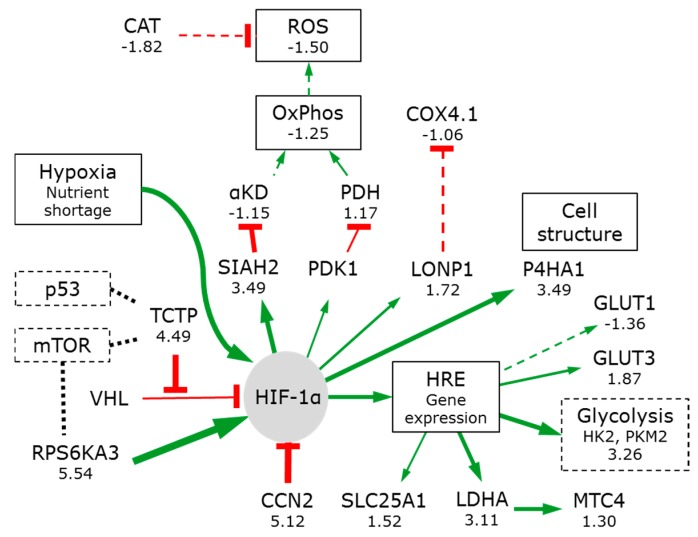
Effects of hypoxia on HIF-1α. The thickness of the line indicates the ratio of protein amount in thre—dimensional (3D) cultures compared to 2D cultures. Dotted lines indicate reduced expression. Green lines ending in arrowheads indicate activators, while red lines ending in a bar indicate inhibitory activity. Dotted-boxes indicate links to other pathway figures.

Hypoxia has numerous effects on mammalian cells. One is the activation of the constitutively expressed hypoxia-inducible factor (HIF-1α) (Figure 4). 

Under normoxia, defined as the PO_2_ levels normally seen in healthy tissues (usually 1–5% [73]), HIF-1α subunits are hydroxylated by prolyl hydroxylases (PHD1-3 including the TCA cycle enzyme α-KD). The modified HIF-1α is recognized and targeted for proteasomal degradation by the VHL-E3-ubiquitin ligase complex.

When oxygen concentrations decrease, the oxygen-dependent PHDs are inactivated, allowing for the HIF-1α protein to accumulate. This promotes HIF-1α translocation to the nucleus where it interacts with HIF-1β/ARNT and p300. This complex binds hypoxia-response elements (HREs) in promoter regions of numerous target genes, including glucose transporters and glycolytic enzymes [82].

The translationally controlled tumour protein (TCTP 4.49) binds competitively to VHL, reducing PHD binding and accelerating HIF-1α accumulation, nuclear translocation, and transcription reprogramming [83].

HIF-1α induces pyruvate dehydrogenase kinase 1 (PDK1) expression. PDK1 inhibits the mitochondrial pyruvate dehydrogenase (PDH) [84]. This reduces pyruvate flux into the TCA cycle and lowers the mitochondrial oxygen requirements. This switch increases lactate production and secretion, as observed by Warburg. Differentially transformed rat embryo fibroblasts showed increasing levels of lactate content and unchanged or decreasing lactate secretion in irrigated spheroids with increasing radii of up to about 450 µM. Above this radius, lactate content and secretion stabilised, illustrating that hypoxia-induced glycolysis need not lead to lactate secretion [58,81] suggesting that most of the glycolytic metabolites are utilised in anabolic processes.

Liver cells can convert lactate back to pyruvate. Despite this, the lactate transporter (MCT4 or SLC16A3) is increased (1.30) suggesting that the cells might ‘pump’ the lactate towards the spheroid surface.

Spheroids show the increase in glucose transporters, glycolytic enzymes and lactate dehydrogenase by on average about a factor of 3.26. This metabolic reprogramming is partial: spheroids do not show a significant decrease in the pyruvate dehydrogenase or of any enzymes of the TCA cycle (Figure 2). 

HIF-1α also induces E3-ubiquitin ligase SIAH2 synthesis. This mediates the proteasomal degradation of the OGDH subunit of α-KD and forms part of the feedback control of HIF-1α. A modest reduction of the α-KD 3 enzyme complex is observed in spheroids (DLD −1.26, DLST −1.11, OGDH −1.09). This will slow the TCA cycle and allow for more citrate to be transported into the cytoplasm by an upregulated citrate transporter protein (SLC25A1, 1.52), supporting the metabolomics [81] and isotope analyses [58,61].

Interestingly HIF-1α also promotes extracellular matrix remodelling via collagen hydroxylases (P4HA1 3.49), a facility useful for cancer cell morphology, adhesion, and motility [85].

Part of the indirect negative feedback regulatory circuit for HIF-1α is the connective tissue growth factor (CCN family member 2) or insulin-like growth factor-binding protein 8, (IBP-8). It is strongly upregulated in spheroids (5.12) illustrating strong positive and negative regulatory mechanisms are active. 

HIF-1α can also induce the mitochondrial protease LONP1, which degrades the less efficient cytochrome C oxidase 4 subunit 1 (COX4-1) from the complex IV of the electron transport chain and allows it to be replaced by the more efficient COX4-2 [82]. Although LONP1 was increased (1.72), there was no change in the level of COX4-1 (−1.06). LONP1 is an essential central regulator of mitochondrial activity and is overexpression in oncogenesis [86]. Despite that spheroids contain higher levels of ATP, subunit IV and the ATP synthase (subunit V) are reduced by −1.12 and −1.37 respectively [18,19]. Reduced mitochondrial respiration will result in fewer reactive oxygen species correlating with reduced levels of catalase (CAT −1.82) [87] resulting in diminishes hydrogen peroxide damage and 50% less oxidised proteins.

### 3.3. Glucose Starvation Has Little Effect on Metabolic Reprogramming

The feature that Warburg noticed—that cancer cells rapidly use glucose and convert it to lactate would suggest that glucose availability might also play a central role.

**Figure 5:**
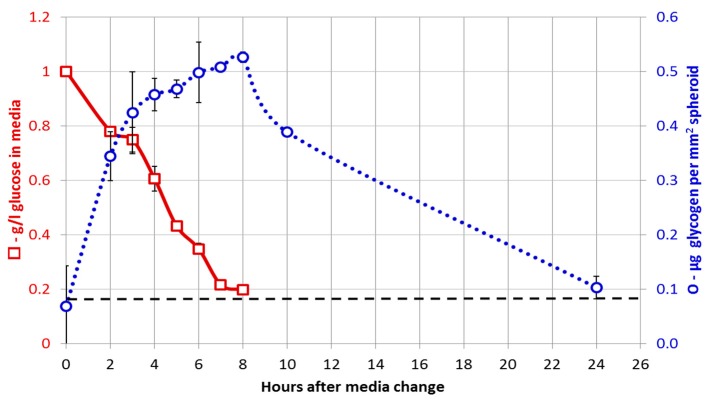
Relationship of glucose consumption from the media with the level of glycogen present in the spheroids. The amount of glucose in the spheroids prior to glycogen hydrolysis was negligible.

Liver cell spheroids are known to rapidly import glucose and convert it to glycogen. When cultured in bioreactors with physiological amounts of glucose (5.5 mM), the media glucose is typically exhausted 8 h after media exchange (Figure 5). Thereafter, the spheroids experience ‘glucose starvation’ and catabolise the glycogen they have synthesised.

Glucose starvation could unleash a number of changes in the cell, as initiated by the Glucose Regulated Proteins (GRP). These are typically found in the ER, often overexpressed in cancers and associated with aggressive growth and invasion [88]. Their first effect would be to increase ER stress and initiate the unfolded (or misfolded) protein response (UPR) [89]. In the UPR, GRP78 dissociates from three protein-folding quality sensors (IRE1, PERK, and ATF6) embedded in the ER membrane. These sensors activate the UPR signal transduction program, a negative feedback loop that alters gene expression to slow protein synthesis and the cell cycle (eventually leading to arrest in G1 [90,91]). Surprisingly, the amount of GRP78 is unchanged between 2D and 3D (1.05) and there are only weak changes in GRP58 (1.34) and GRP60 (1.37), suggesting that there is no UPR or ER stress. 

The mitochondrial GRP75 can inactivate p53 and induce apoptosis [88], but it is only slightly elevated (1.36), suggesting that the mitochondria also suffer very little stress.

GRP94 and GRP170 show the strongest responses (2.09 and 2.15, respectively). GRP 94 plays critical roles in folding and exporting proteins in the secretory pathway (e.g., insulin-like growth factors IGF-1 and 2), which could activate the PI3K-Akt pathway. GRP170 is a glycosylated protein also known as the hypoxia up-regulated protein 1, HYOU1. It plays a role in suppressing apoptosis and is up-regulated in invasive tumours [88]. Considering the relatively little stress caused by prolonged glucose starvation, the lack of glucose appears to play a minor role in the metabolic reprogramming. What effects there are, appear to stabilise cellular metabolism and are anti-apoptotic.

### 3.4. Metabolic Reprogramming ‘Links’ Glutamine Metabolism to the Hexosamine Pathway

Metabolic reprogramming results in an increased reliance on glutamine. Intracellular levels are regulated by plasma membrane transporters SLC1A5 and SLC38A2 [92]. ER stress would induce their degradation and ultimately to autophagy and cell death [92,93]. In spheroids, SLC38A2 is increased (1.96) while SLC1A5 is decreased (−1.29), suggesting that they play subtly different roles. In agreement with this, net glutamine uptake in HeLa cells was not dependent on SLC1A5 but required SLC38A1 or 2 [94].

#### 3.4.1. Conversion of Glutamine to Glutamate

The cells’ glutamate demand is probably supplied by the highly upregulated GFPT1 which is the first, and rate-limiting step, of the hexosamine pathway (8.37). This enzyme catalyses the conversion of fructose 6-phosphate and glutamine to glucosamine 6-phosphate and glutamate. Activation of glutaminolysis was necessary for adaptive cell survival in the mouse model of pancreatic ductal adenocarcinoma [95]. Hypoxia is considered to drive this adaptive process, which, amongst other things, leads to increased amounts of O-linked N-acetylglucosaminylated proteins. In agreement with this, several polysaccharide, proteoglycan and glycosylation synthetic pathway enzymes are strongly upregulated in 3D spheroids (UDP-glucose pyrophosphorylase UGP2 6.59; UDP-glucose 6-dehydrogenase UGD 7.46; UDP-glucose 4-epimerase GALE 12.71; and sialic acid synthase NANS 5.37).

#### 3.4.2. α-Ketoglutarate

Glutamate can be converted to α-ketoglutarate by the mitochondrial GLS1 (−1.47) and GLUD (1.19). It can also be converted by cytoplasmic or mitochondrial alanine or aspartate aminotransferases [50] and it is the cytoplasmic enzyme that is upregulated (GOT1 1.91, GOT2 −1.34).

There are three possible routes by which α-ketoglutarate can be converted to citrate (Figure 2). Firstly, it can be converted around the TCA cycle. Secondly it could be converted via isocitrate to citrate (by IDH2 1.40 and ACO2 1.67), reversing the normal TCA cycle flux by reductive glutamate metabolism [61]. IDH3 is not increased (1.08) because it can only catalyse the ‘forward’ reaction. Finally cytoplasmic α-ketoglutarate, produced via the upregulated GFPT1 and GOT1, can be converted, by IDH1 (1.90) and ACO1. While all three processes probably occur, both isotope tracing and enzyme abundance suggests that the latter route is the most active [57,61].

#### 3.4.3. NADH

Conversion of α-ketoglutarate to isocitrate requires the cofactor NADPH. The reduction in the mitochondrial MDH2 (−1.35) and the essentially unchanged abundance of its NAD(P) transhydrogenase (NNT 1.13) suggest that the mitochondrial source is of low significance. MDH1 is increased (1.53) but lacks a malate source (the SLC25A11 transporter is reduced −1.28) and the conversion of cytoplasmic pyruvate to lactate would actually consume the NADH that is produced. The richest source of NADPH is the pentose phosphate pathway where G6PD and 6PD are both upregulated (1.90 and 2.37 fold respectively), in agreement with isotope tracing data [57,61].

#### 3.4.4. Citrate

Citrate is used for fatty acid synthesis. ATP-citrate synthase (ACL 2.94) uses citrate to generate cytosolic acetyl-CoA. Acetyl-CoA is used for: histone acetylation by acetyl-CoA acyltransferase (ACAA1 7.66); palmitate synthesis by fatty acid synthase (FASN 2.76); cholesterol, steroid hormones, haem and a plethora of other biomolecules. Glutamine is as important as glucose in metabolic reprogramming and blocking glutamate-dependent cellular pathways (at either IDH1 or ACL) limits tumorigenic growth [49,58].

### 3.5. Metabolic Reprogramming Is Associated with Chromatin Remodelling

Conversion between transcriptionally active euchromatin and inactive heterochromatin is brought about by processes, including acetylation, methylation, and clipping of histones. Hypoxia can change these epigenetic markings. HIF-1α stabilisation leads to increases in histone lysine demethylases (KDM3A, KDM4B, KDM4C, and KDM6B) [96]. While C3A cells that are grown in 2D culture essentially show little epigenetic marking, spheroids recover extensive histone methylation, acetylation, and clipping on both H2B and H3 [97]. Hypoxia also upregulates the arginine *N*-methyltransferase PRMT1 (2.66), increasing methylation of arginine 3 of H4 [98]. PRMT1 can asymmetrically methylate the ReIA subunit [99] inhibiting its binding to DNA and repressing NF-κB target genes. The ‘Chromatin target of PRMT1’ protein, (CHTOP) [100] which promotes cell cycle progression, is strongly reduced in spheroids (−8.09), resulting in few cells in the G2/M phase. Histone deacetylases do not appear to be affected (HDAC1 1.00).

### 3.6. The Switch to Anabolic Metabolism

Spheroids composed of either OVTOKO or SIHA cell lines have been shown to contain higher levels of serine, glutamine and other amino acids as well as citrate [81]. The amounts of all anabolic rate-limiting enzymes are increased while catabolic enzymes are unchanged (Table 1) in concordance with metabolic reprogramming to aerobic glycolysis. 

The three rate-limiting glycolytic pathway steps (HK2, PFKL, and PKM) are three of the four most increased enzymes of the pathway (the 4th being aldolase). Interestingly, PFKL is repressed by high ATP/AMP ratios [101]. Since spheroids have high ATP amounts [19], high PFK levels suggest that AMP levels are also high.

The glutamine-dependent cytosolic carbamoyl-phosphate synthetase 2, is upregulated in spheroids (CAD 3.49). CAD is the rate-limiting enzyme carrying out the first three steps in pyrimidine synthesis. CAD is essential for uridine diphosphate (UDP) synthesis, which in turn, is essential for glycogenesis. This correlates with the appearance of glycogen granules in hepatocyte spheroids and with protein glycosylation and the hexosamine pathway [102].

### 3.7. Signal Pathways Involved in Orchestrating Metabolic Reprogramming

All of the adaptations seen in glycolysis and glutaminolysis, pentose phosphate pathway, TCA cycle, and fatty acid synthesis indicate that spheroids, grown in a wide variety of 3D culture systems, are utilising a significant degree of metabolic reprogramming to aerobic glycolysis.

The typical features of 3D culture—diffusion gradients resulting in hypoxia (and to a less extent glucose starvation) clearly drive metabolic reprogramming. Warburg saw this phenomenon as a hallmark of cancer. In order to investigate how metabolic reprogramming is orchestrated, we reviewed the status of pathways that are often associated with tumour development: PI3K/Akt/mTOR, Myc, p53, nuclear factor kappa-B (NF-κB), and Wnt [54].

#### 3.7.1. PIK3/AKT/mTOR

**Figure 6:**
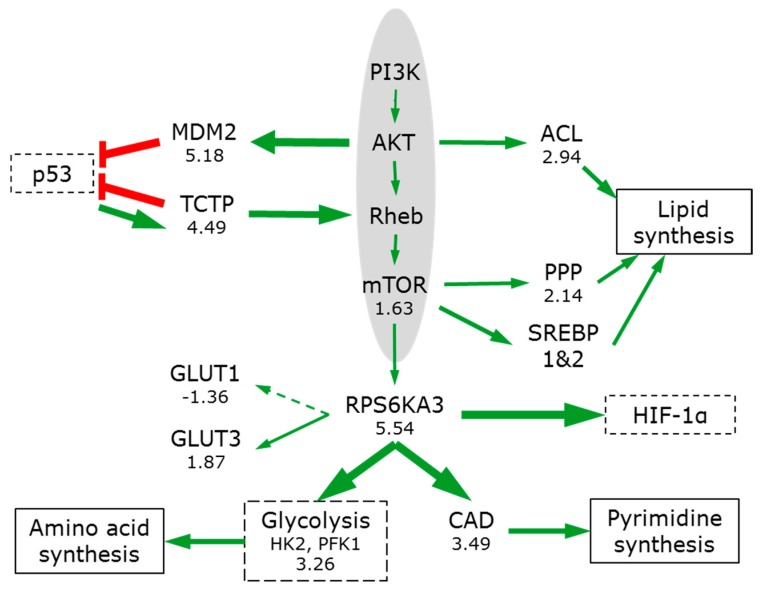
mTOR signalling in 3D spheroids. See legend to Figure 4 for nomenclature.

The PI3K/AKT/mTOR pathway (Figure 6) plays a key integrating role, sensing concentrations of nutrients (including glucose, oxygen, amino acids and ATP levels) and regulating the anabolic processes of the cell for growth and maintenance [103].

While only two key proteins from this pathway were detected (mTOR, 1.63; and ribosomal protein S6 kinase RPS6KA3, 5.54), strong downstream effects are clearly visible showing that pathway is activated in 3D (Table 1). mTOR signalling increases translation of hypoxia-inducible factor 1α (HIF-1α), glucose transporters and glycolytic enzymes, and promotes metabolic reprogramming [101,104] (Figure 6).

mTOR promotes pentose phosphate pathway (PPP) enzyme expression (on average by 2.14) and channels metabolic flux into its oxidative, NADPH-producing branch [91]. mTOR strongly stimulates pyrimidine synthesis via the RPS6KA-mediated phosphorylation of CAD (3.49), thereby increasing the pool of nucleotides available [105]. AKT can phosphorylate ACL, enhancing its lipogenic activities and mTOR signalling promotes NADPH-requiring lipid synthesis by activating sterol regulatory element-binding proteins (SREBP1 and 2) [106].

#### 3.7.2. Myc

**Figure 7:**
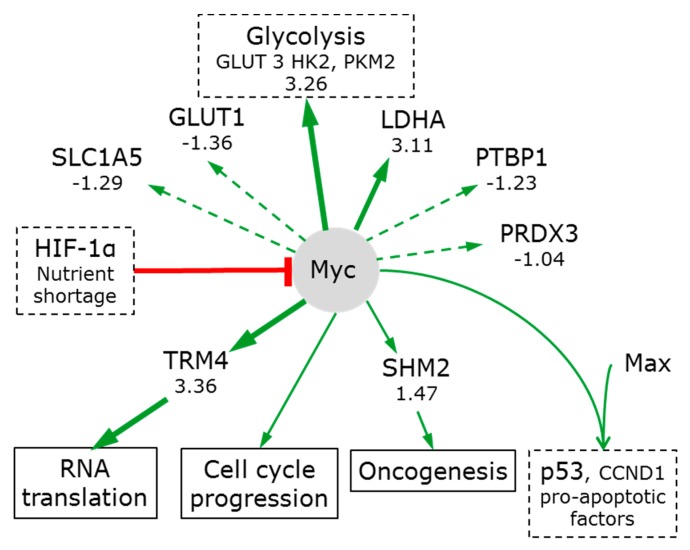
Myc signalling in 3D spheroids. See legend to Figure 4 for nomenclature.

Myc has the potential to play a key role in metabolic reprogramming. Myc is central to growth regulation and is one of the most frequently deregulated oncogene transcription factors seen in a wide variety of cancers [107,108]. Myc directly transactivates gene expression of GLUT1, phosphofructokinase (PFK), enolase (ENO) and LDHA and indirectly increases phosphoglucose isomerase (GPI), glyceraldehyde-3-phosphate dehydrogenase (GAPDH) and phosphoglycerate kinase (PGK1) [109] (Figure 7). This is consistent (with the exception of GLUT1) with their increased levels in spheroids. However, as described above, PIK3/AKT/mTOR can also induce these proteins (via HIF-1α) and so this effect need not be attributed to Myc. HIF-1α can inactivate Myc [110], and in doing so, induce cell cycle arrest [111].

Low expression levels of several proteins normally induced by Myc suggest that Myc is not particularly active in 3D spheroids. Examples include: PTBP1 [112] (−1.23); GLUT1 (−1.36); SLC1A5 [107] (−1.29); and, PRDX3’s [113] (−1.04). Myc regulates serine hydroxymethyl transferases and pathway hyperactivation is a driver of oncogenesis [107]. However, the moderate increase of SHMT2 (1.47) cannot qualify as hyperactivation. One exception may be tRNA (cytosine34-C5)-methyltransferase (TRM4 which methylates the first position of the cytosine anticodon). Myc enhances TRM4 expression (3.36). The formation of a covalent complex between dual-cysteine RNA:m5C methyltransferases and methylated RNA has been proposed to provide a unique mechanism by which metabolic factors can influence RNA translation, in particular the processing and utilisation of m5C-containing RNAs [114]. 

Nutrient shortage and/or hypoxia can inhibit Myc translation; reduce its stability and its ability to dimerise with another transcription factor MAX. Inhibition of Myc/MAX dimerization prevents specific gene expression, most significantly of p53, cyclin D1 and pro-apoptotic factors [115]. Therefore, while Myc regulates many proteins in cancer [107], it appears that the slow proliferation of cells in spheroids is a result of low myc activity.

#### 3.7.3. p53

The tumour suppressor p53 can transactivate a broad array of target genes that are involved in redox maintenance, DNA repair, cell cycle checkpoints, and can thus affect cellular senescence, proliferation, and apoptosis. Mutations of p53 are found in over 50% of human tumours and disturb the IGF1-AKT branch of the mTOR pathway [116].

**Figure 8:**
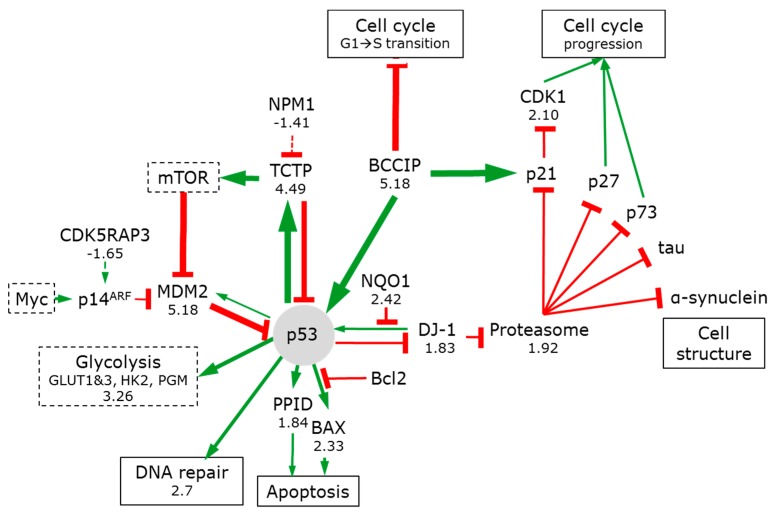
p53 signalling in 3D spheroids. See legend to Figure 4 for nomenclature.

p53 activity is tightly linked to the oncogene protein DJ-1 (1.83). In a self-regulating loop, DJ-1 is necessary for hypoxic stress-induced p53 activation, while p53 prevents the accumulation of the DJ-1 protein (Figure 8). DJ-1 can bind the ubiquitin-independent 20S proteasomal core and its quantitative increase mirrors the increase in the core (1.92) and in NADPH:quinone oxidoreductase 1 (NQO1, by 2.42) (which protects p53 from proteasomal degradation).

Many key regulatory proteins, including tumour suppressors p53 and p73, tau, α-synuclein and the cell cycle regulators p21 and p27 are degraded by the proteasome core. DJ-1 binding inhibits the activity of the core and by slowing their degradation, leads to an increase in their abundance and activity [117]. DJ-1 can also activate the AKT/mTOR pathway [116].

In addition, the transcriptional suppressor CDK5RAP3 is reduced (−1.65). This will allow for the synthesis of p14ARF and its binding to MDM2. This releases p53 from inhibition. This results in the stabilization, accumulation, and activation of p53 [118].

Activation of p53 is consistent with the increase in DNA repair enzyme expression [57] (on average by 2.7). Interestingly, both the positive (BCCIP) and negative (TCTP) p53 regulators are strongly increased (5.18 and 4.49, respectively), illustrating that p53 is subjected to a tight feedback regulation. BCCIPβ plays a role in cell growth regulation [119]. Overexpression of the BCCIPβ splices variant delays the G1-to-S cell cycle transition and elevates p21 expression. Elevated p21 expression would inhibit cyclin dependent kinase 1 (CDK1 2.10) induction of cell cycle progression.

The evolutionarily conserved TCTP is emerging as a pleiotropic key to phenotypic reprogramming through its ability to regulate the mTOR pathway [120], as well as being an upstream activator of OCT4 and NANOG transcription factors (which play essential roles in nuclear reprogramming). p53 induces TCTP, reducing oxidative stress and minimizing apoptosis [121]. Forming another negative feedback loop, TCTP can inhibit both transcription and function of p53 [122]. The activation of TCTP (4.49) suggests reduced proliferative drive [123]. This is confirmed by the reduction in nucleoplasmin (NPM1 −1.41), which would otherwise complex with TCTP during mitosis to promote cell proliferation.

p53 directly regulates cellular redox homeostasis by modifying expression of pro- and anti-oxidant enzymes peroxiredoxins and thioredoxins. Peroxiredoxins (that act as both sensors and barriers to MAPK activation) are upregulated (PRDX1-6 of 1.74, 2.27, −1.04, 1.86, 1.42, and 2.75, respectively), as is thioredoxin (TXN 1.84). Their upregulation contributes to Myc regulation [124]. The modest changes in mitochondrial peroxiredoxins (PRDX 3 and 5: −1.04 and 1.42) suggest that mitochondrial ROS are insignificant ‘stress factors’ in keeping with the relatively reduced mitochondrial activity.

p53 is activated, but is exposed to tight feedback control. Together with the low activity of Myc, p53 and associated pathways arrest the cells predominantly in G1 or Go [19].

#### 3.7.4. Wnt GSK-3β/β-Catenin

In the canonical Wnt pathway, the Wnt ligand can bind to a Frizzled family receptor, causing a deactivation of the β-catenin destruction complex. This leads to the dephosphorylation of β-catenin, its accumulation and migration to the nucleus where it acts as a coactivator of TCF/LEF transcription factors. Activation of the Wnt/β-catenin pathway activates cell proliferation and the homeostatic renewal of the liver from pericentral hepatocytes [125]. In 3D spheroids, this pathway is inactive: the amount of β-catenin is reduced (CTNNB1 −1.57), and the protein phosphorylase 2A, although present (PPP2RA1 (the constant regulatory subunit core of the PP2A) 1.02), is strongly inhibited by I1PP2A and I2PP2A (5.77 and 2.18). mTOR also negatively regulates PP2A, allowing for the integration of these two pathways. Reverse regulation occurs in amino-acid depleted conditions: PP2A can inhibit mTOR via dephosphorylation of p170 [126].

The histidine triad nucleotide-binding protein 1 is significantly increased (HINT1, 5.99). It keeps the Wnt GSK-3β/β-catenin pathway inactive, limits cell growth [127], and promotes apoptosis via p53 and Bax [128]. The mitochondrial HINT2 may also promote apoptosis (1.32).

In contrast, the non-canonical Wnt pathway appears to be active in 3D spheroids. Binding of Wnt to Frizzled recruits Dsh, which then binds directly to RAC1 (1.40) and indirectly to profilin (2.75) amongst others. Both of these and numerous other upregulated actin-structure modifying proteins lead to the dramatic restructuring seen in spheroids [57].

#### 3.7.5. NF-κB

NF-κB is a rapid-acting primary transcription factor well suited to respond to harmful stimuli like cell stress, cytokines and free radicals. Many different types of human tumours have constitutively active NF-κB [129].

In its inactive state, the NF-κB heterodimer (composed of p50 and ReiA) is complexed with its inhibitor IκBα. In spheroids, HINT1 (5.99) promotes IκBα stability maintaining NF-κB inactive [127]. Hypoxia upregulates PRMT1 (2.66) [57], which asymmetrically methylates ReIA inhibiting ReIA’s binding to DNA, and further repressing NF-κB [99]. The type III transforming growth factor β receptor (TGFβR3) regulates both the epithelial-mesenchymal transition and cell invasion during development via NF-κB activation [130]. Deactivation of TGFβR3 (−4.58) is consistent with NF-κB inactivity. The extracellular matrix TGFβ-induced protein (TGFBI), involved in tissue remodelling and found in liver metastases stroma, is very highly upregulated (TGFBI 8.83) [131]. TGFBI reduces NF-κB activation [132]. Deactivation of NF-κB in 3D sensitises the cell to apoptosis or necrosis by allowing for TNF-α to active the JNK pathway and lead to cell death.

#### 3.7.6. Cell Death

Many of the pathways described above influence necrosis and apoptosis. The fundamental difference between them is that bioenergetic failure in necrosis leads to free radical damage, swelling, rupture, and cytolysis, while apoptosis is ATP-requiring and leads to shrinkage, caspase activation, DNA fragmentation, and retention of the plasma membrane [133]. 

Apoptosis is often ‘defeated’ as a cell is transformed from healthy to tumourigenic. Many specific mechanisms operating in many organelles can lead to apoptosis [133]. The apoptotic potential is a balance between pro- and anti-apoptotic signals, which are integrated in mitochondria. The decreased amounts of NF-κB and other factors noted above, result in the under expression of anti-apoptotic proteins including Bcl-2; Bcl-XL; NR13; Bcl-2 inhibitor of transcription 1 (PTRH2 −1.37); Bcl-2-associated transcription factor 1 (BCLAF1 −2.61); Bcl-2-associated athanogene 2 (BAG2 −1.18); Bcl-XL-binding protein v68 (PGAM5 −1.78) and the ‘defender against apoptotic cell death’ (DAD1 −1.37). These anti-apoptotoic proteins would otherwise bind and inactivate pro-apoptotic proteins. The only pro-apoptotic protein detected, BAX (Bcl-2-like protein 4), was increased (2.33). The net result in spheroids is to increase their apoptotic sensitivity, but without activating apoptosis.

Necrosis, as judged by the microscopic appearance of core cells, has often been reported for spheroids. Activated p53 would interact directly with PPID and push the cell towards necrosis [134]. This interaction may be enhanced by increased BAX abundance (2.33), especially when anti-apoptotic Bcl2 proteins are depleted. Thus, both apoptotic and necrotic processes are sensitised.

3D spheroid cultures have illustrated that the serine protease tumour suppressor MASPIN facilitates the mitochondrial permeability transition (MPT). However, since ATP levels are high, neither process opens the MPT pore. Its opening would initiate a collapse of the transmembrane proton gradient and lead to apoptotic or necrotic cell death (depending on the initiating factors). The essential component of the MPT pore, the peptidyl-prolyl isomerase D, located in the mitochondrial matrix is increased (PPID 1.84). The non-essential components, VDAC, (Voltage Dependent Anion Channel, which spans the outer membrane) and ANT (Adenine Nucleotide Translocase which spans the inner membrane) are either unchanged or are decreased (VDAC1 1.02; VDAC2 −1.16; VDAC3 −1.23; ANT1, (ATP/ADP antiporter SLC25A4) −1.01; ANT2, −1.98; ANT3 −1.25). ANT1 can interact with BAX. ANT2 is anti-apoptotic and it’s reduction matches other anti-apoptotic BCl-2 proteins. On the balance, necrosis may be favoured over apoptosis due to the reduction in the chromatinolytic activity of AIFM1 (apoptosis-inducing factor mitochondrion-associated 1, −1.90) [135].

## 4. Conclusions

The most widely used approach to reproducibly produce 3D spheroids or organoids that are stable for long periods of time are clinostat ‘microgravity’ cultures in micro-bioreactors. In these spheroids, the majority of cells experience hypoxia and glucose starvation. These conditions are certainly closer to those present in tissues than those experienced by cells in classical 2D cultures (which typically experience hyperoxia and hyperglycaemia), and are therefore critical to take into account when designing a micro-bioreactor. The recovery of physiological behavior stems from:

1. Oxygen limitations (and to a less extent glucose) induce metabolic reprogramming from oxidative phosphorylation to aerobic glycolysis and result in a strong anabolic phenotype.
2. The metabolic reprogramming includes an activation of glutaminolysis (via extra-mitochondrial pathways) (consistent with physiological increases in lipid and cholesterol synthesis).
3. Glutamine conversion to the lipid ‘precursor’ glutamate is linked to the hexosamine pathway activation. This correlates to increased glycogen production and protein glycosylation.
4. The additional NADPH needed for citrate and lipid synthesis is mainly generated by pentose phosphate pathway activation. Increases in acetyl-CoA also provide precursors for the observed histone acetylation.
5. Signalling pathway activities (activation of mTOR and p53, repression of NF-κB and canonical Wnt) are consistent with significant retardation of proliferation and the accumulation of cells in G1/G0, (resulting in a rate resembling that seen in both healthy and transformed cells in tissues and tumours).
6. The reduction in proliferation rate allows the cell to achieve higher ATP levels.
7. Activation of the non-canonical Wnt signalling pathway orchestrates the significant ultrastructural changes.
8. The rate of proliferation is not coupled to aerobic glycolysis.
9. Metabolic reprogramming underpins the recovery of traits mimicking in vivo physiology.

3D tissues offer an exciting model to investigate in vivo-like functionality where cells are grown in conditions that are not drastically different to those seen in vivo. Given the right growth conditions, cells ‘spontaneously’ revert to an in vivo mimetic physiological performance.

## Figures and Tables

**Table 1:** Rate limiting enzymes for central metabolic pathways and the ration of their expression in 3D spheroids compared to 2D exponential growth. n.d. not detected.

Pathway	Gene	Fold Change
Glucose phosphorylation	*HK2*	2.81
Glycogenolysis	*PYGB*	4.06
Glycolysis	*PFKL*	5.43
Glycolysis	*PKM*	3.21
Pentose Phosphate	*G6PD*	1.90
Hexose	*GFPT1*	8.37
TCA Cycle	*IDH2 & 3*	1.40 & 1.08
Pyrimidine synthesis	*CAD*	3.49
Purine synthesis	*PRPS1*	3.74
Fatty acid synthesis	*FASN*	2.76
Fatty acid synthesis	*ACAA1*	7.66
Fatty acid oxidation	*CRAT*	1.17
Alanine synthesis	*ALT*	n.d.
Asparagine synthesis	*ASNS*	5.58
Aspartate synthesis	*GOT1*	1.91
Cysteine synthesis	*MAT1*	5.62
Glutamine-glutamate conversion	*GLUD*	1.19
	*GFPT1*	8.37
Glycine synthesis	*SHMT*	1.47
Methionine synthesis	*MTR*	n.d.
Proline synthesis	*PYCR1* & *2*	1.06 & 1.03
Serine synthesis	*PHGDH*	7.67
Tyrosine synthesis	*PAH*	3.80
Urea synthesis	*CPS*	3.49
Folate synthesis	*MDHFD1*	2.49

## Supplementary Materials

The following are available online at http://www.mdpi.com/2306-5354/5/1/22/s1, Video S1: 17 day old spheroids in clinostat culture.

Supplementary File 1
